# Crystal structure and analysis of HdaB: The enteroaggregative *Escherichia coli* AAF/IV pilus tip protein

**DOI:** 10.1002/pro.2982

**Published:** 2016-08-01

**Authors:** Wei‐Chao Lee, Steve Matthews, James. A. Garnett

**Affiliations:** ^1^ Department of Life SciencesCentre for Structural Biology, Imperial College LondonSouth KensingtonLondonSW7 2AZUnited Kingdom; ^2^ School of Biological and Chemical SciencesQueen Mary University LondonLondonE1 4NSUnited Kingdom

**Keywords:** AAF/IV, HdaB, chaperone‐usher, adhesion, invasion, pilus, fimbria, *Escherichia coli*

## Abstract

Enteroaggregative *Escherichia coli* is the primary cause of pediatric diarrhea in developing countries. They utilize aggregative adherence fimbriae (AAFs) to promote initial adherence to the host intestinal mucosa, promote the formation of biofilms, and mediate host invasion. Five AAFs have been identified to date and AAF/IV is amongst the most prevalent found in clinical isolates. Here we present the X‐ray crystal structure of the AAF/IV tip protein HdaB at 2.0 Å resolution. It shares high structural homology with members of the Afa/Dr superfamily of fimbriae, which are involved in host invasion. We highlight surface exposed residues that share sequence homology and propose that these may function in invasion and also non‐conserved regions that could mediate HdaB specific adhesive functions.

## Abstract 2

PDB Code(s): 5D55

**Abbreviations**
AA: aggregative adherence
AAF: aggregative adherence fimbriae
EAEC: enteroaggregative *E. coli*
HUS: hemolytic uremic syndrome
NMR: nuclear magnetic resonance
Stx: Shiga toxin

## Introduction

*Escherichia coli* is a Gram‐negative bacterium that colonizes the bowels of humans and other animals. Although the majority of strains have developed a commensal relationship with their host, several *E. coli* strains are highly pathogenic and harbor virulence factors to promote biofilm formation, evasion of host immune responses to infection, and ultimately cause severe illness and death. Enteroaggregative *E. coli* (EAEC) is the primary cause of pediatric diarrhea in developing countries^1^, ^2^ and its defining characteristic is an aggregative adherence (AA) pattern to HEp‐2 cells *in vitro*,^1^ which appear as a stacked brick‐like arrangement of adherent bacteria. In 2011 a Shiga toxin (Stx)‐producing strain of EAEC was responsible for a large outbreak in Germany, which spread across Europe and resulted in 3816 cases of gastroenteritis, 845 cases of hemolytic uremic syndrome (HUS), and 54 fatalities.^3^, ^4^, ^5^ This O104:H4 strain was significantly more infectious than other Stx‐producing *E. coli* strains because of its specific arsenal of EAEC virulence factors, including aggregative adherence fimbriae (AAFs).^3^

AAFs are essential EAEC factors that promote initial adherence to the host intestinal mucosa, promote the formation of biofilms but can also mediate host invasion.^6^, ^7^, ^8^ Four variant AAFs have been characterized to date (AAF/I to AAF/IV) and a new one has also been recently identified (AAF/V).^9^, ^10^, ^11^, ^12^, ^13^, ^14^ These are located on a 55–65 MDa plasmid (pAA) and are encoded by the *agg* (aggregative), *aaf* (aggregative adherence fimbriae)*, agg3* (aggregative 3), *hda* (HUS‐associated diffuse adherence), and *aaf5* (aggregative adherence fimbriae 5) gene clusters, respectively.

AAFs are assembled via the FGL chaperone/usher (CU) pathway.^8^, ^15^, ^16^, ^17^ CU systems are composed of an outer membrane ‘usher’ pore and usually a single chaperone and several fimbrial/pilin domains. Pilin domains form the final polymeric structure and are composed of an Ig‐like fold that lacks the final G‐strand, but instead this is presented as an unstructured N‐terminal extension (NTE). Upon entry into the periplasm these domains form a complex with the chaperone, which stabilizes them, prevents their auto‐polymerization and directs them to the usher. At the outer‐membrane the NTE of one pilin domain is inserted into an adjacent pilin domain, completing the Ig‐like fold and as the fiber polymerizes it is secreted through the usher pore into the extracellular space.

EAEC strains express one or more AAF structures and although functional redundancy exists, there is evidence that AAFs also perform specialized roles.^18^ The structures of AAF/I and AAF/II were recently resolved and are composed of a major subunit (AggA and AafA, respectively), serving as the chief polymeric unit, and a single minor capping subunit that lacks the NTE (AggB and AafB, respectively).^8^ The major subunits have significant positive charge and mediate electrostatic interactions with host receptors including fibronectin, although they have only low structural homology with one another. The minor subunits, however, share a conserved tertiary arrangement and are also highly similar to members of the Afa/Dr superfamily, which are responsible for the recruitment of host integrin and cellular invasion.^19^, ^20^

In this study, we report the X‐ray crystal structure of the AAF/IV pilus tip subunit, HdaB. This is a donor strand complemented construct (HdaB‐dsA) and represents the structure of the HdaB domain in the final AAF/IV fiber. Here the first 10‐residues (NTE) of the AAF/IV major subunit (HdaA) are fused after an artificial linker sequence to the C‐terminus of HdaB. AAF/IV is amongst the most abundant AAF structure identified in EAEC clinical isolates^13^, ^14^ and we show that HdaB too shares structural homology with members of the Afa/Dr superfamily and other FGL CU assembled pilin subunits. Primary and tertiary structure analyses of these proteins with HdaB highlight potential regions involved in host invasion but also fiber‐specific carbohydrate recognition. Finally, our structure of HdaB‐dsA is formed through an artificially induced covalent domain‐swapped dimer. A cysteine residue from within the linker forms a disulfide bond between the subunits and this could be used as a strategy to obtain crystals in similar systems where it has not previously been possible.

## Results and Discussion

### Overall structure

**Figure 1:**
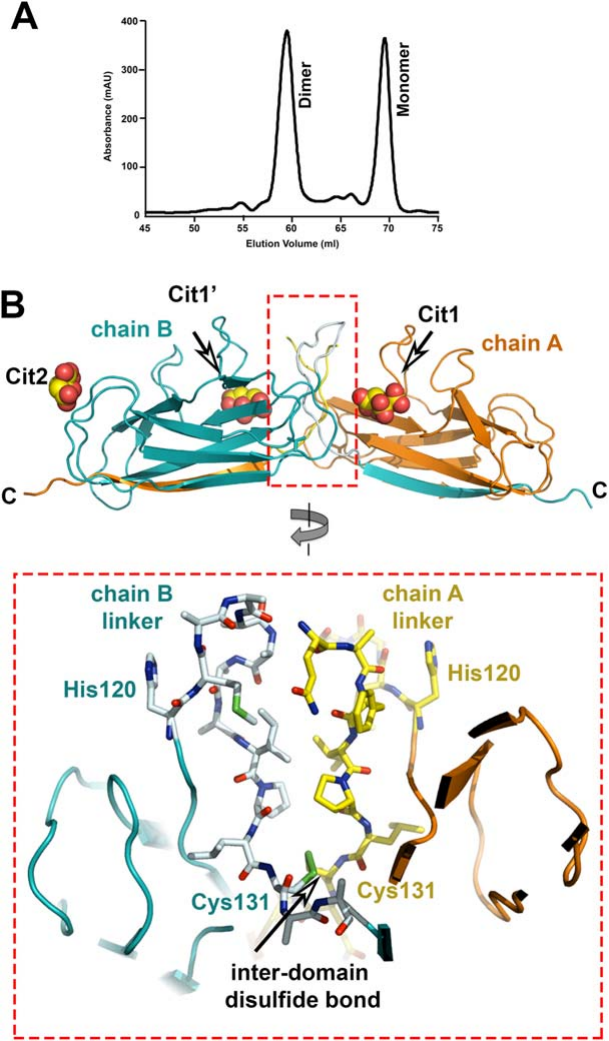
The HdaB‐dsA domain‐swapped dimer. A: Gel filtration profile of monomeric (17.5 kDa) and dimeric (35 kDa) HdaB‐dsA. B: Asymmetric unit of HdaB‐dsA crystals. Upper panel: domain‐swapped dimer of HdaB‐dsA shown as cartoon with citrate ions shown as spheres. The linker region is boxed and expanded below. Lower panel: the linker regions shown as sticks and also the Cys131‐Cys131 inter‐domain disulfide bond are highlighted.

To create HdaB‐dsA, the N‐terminal donor strand of HdaA (residues 1‐10) was fused to the C‐terminus of HdaB (residues 1‐119), with an intervening 14‐residue linker (HMDNKQEFIPLCQA). During purification of HdaB‐dsA two bands eluted during gel filtration, corresponding to a monomer and dimer [Fig. 1(A)]. Both forms were used to set up crystallization trials, yet only the dimeric species successfully crystallized.

The structure of HdaB‐dsA was determined by molecular replacement and refined to 2 Å resolution (Table 1). Due to the high structural homology reported between AggB and AafB, the coordinates of AafB were used as the search model (47% sequence identity).^8^ HdaB‐dsA crystals belong to *P4_3_2_1_2* space group and the asymmetric unit consists of two molecules composed of a domain‐swapped dimer [Fig. 1(B)]. Here the HdaA donor strand from one subunit is inserted into the acceptor groove of its dimer mate. However, in addition to the conserved intra‐domain disulfide bond (Cys28‐Cys117) an inter‐domain disulfide bond (Cys131‐Cys131) is also formed between the synthetic linker from each chain, which loop out, fold back against the C‐terminus of HdaB and pack against one another [Fig. 1(B)]

**Table 1:** Crystallographic Data and Refinement Statistics for HdaB‐dsA

	
**Crystal parameters**	
Space group	*P4_3_2_1_2*
Cell dimensions (Å)	*a* = *b* = 112.2940, *c* = 61.6521
Number of protein molecules per asymmetric unit	2
**Data Collection**	
Beamline	DLS I24
Wavelength (Å)	1.65310
Resolution (Å)	28.74‐2.00 (2.11‐2)
Unique observations	4,82,265 (27,204)
*R*_merge_	0.371 (0.054)
< *I* >/σ*I*	5.4 (1.9)
Completeness (%)	98.1 (95.1)
Redundancy	3.6 (3.1)
Wilson *B* value (Å^2^)	31.6
Average *B* value (Å^2^)	34.1
**Refinement**	
*R*_work_/*R*_free_ (%)	20.2/24.8
Number of protein residues in the asymmetric unit	294
Number of ligands/ions	3 citrates, 1 iodide ion
Number of water molecules	318
**Rmsd stereochemistry**	
Bond length (Å)	0.009
Bond angles (°)	1.184
**Ramachandran analysis**	
Residues in favored regions	97.7%
Residues in allowed regions	100%

Numbers in parentheses refer to the outermost resolution shell.

*R*_merge_ = Σ|*I* – |/Σ*I* where *I* is the integrated intensity of a given reflection and is the mean intensity of multiple corresponding symmetry‐related reflections.

*R*_work_ = Σǁ*F*_o_| – |*F*_c_ǁ/Σ*F*_o_ where *F*_o_ and *F*_c_ are the observed and calculated structure factors, respectively.

*R*_free_ = *R*_work_ calculated using ∼10% random data excluded from the refinement.

Rmsd stereochemistry is the deviation from ideal values.

Ramachandran analysis was carried out using Molprobity^21^.

Therefore this dimer is in fact a covalent one, albeit artificially induced, with a significant proportion of its interface (∼8000 Å^2^) provided by the large linker; which is further stabilized by the inter‐domain disulfide bond. Although several domain‐swapped oligomeric structures have been reported for other CU assembled pilin domains,^22^, ^23^, ^24^ the inter‐domain disulfide bond observed here is unique to HdaB‐dsA. As it was not possible to obtain crystals for monomeric HdaB‐dsA, formation of this dimer was essential for the successful structure elucidation of HdaB‐dsA. Therefore introduction of such an extended linker containing a cysteine at this specific site could be used to promote crystallization of other proteins from similar systems.

**Figure 2:**
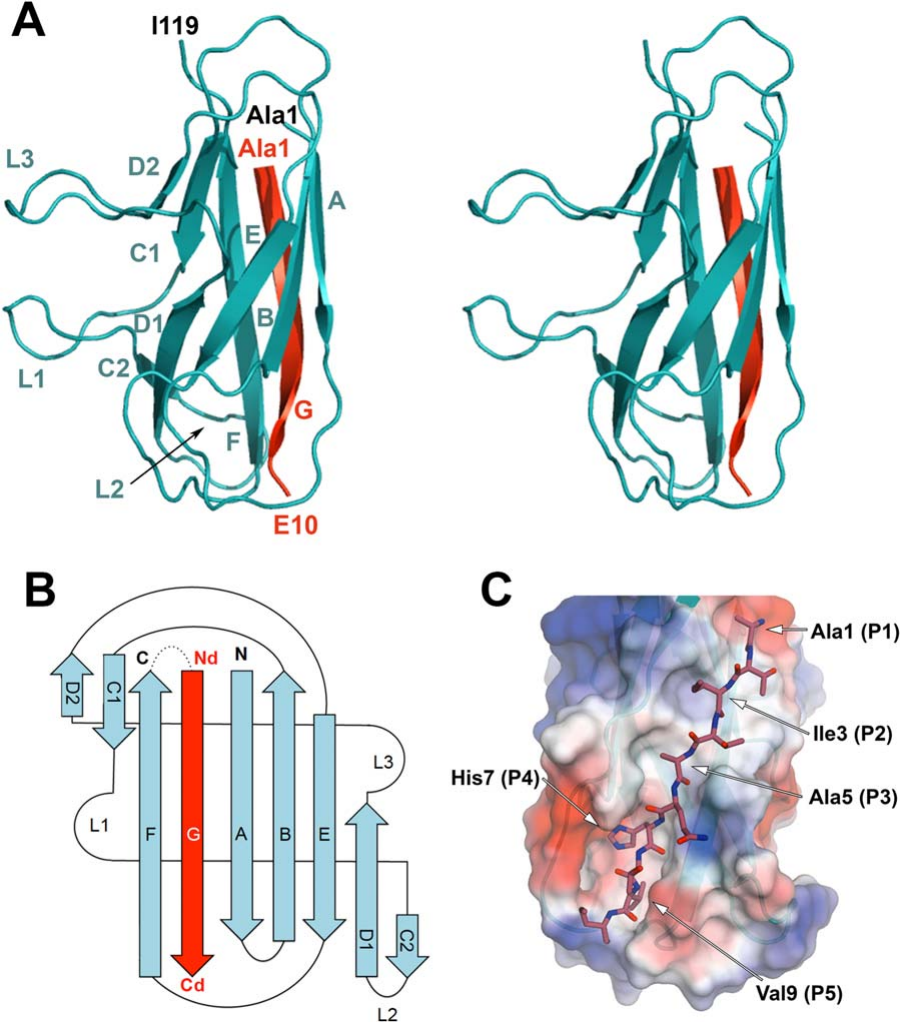
Overall Structure of HdaB‐dsA. A: Stereo cartoon representation of an individual HdaB‐dsA monomer with secondary structure labeled (β‐strands and loops). HdaB from chain A is colored teal whilst the HdaA donor strand from chain B is colored red. N/C‐termini are annotated as residue type/number in red (HdaA) and black (HdaB). For clarity the artificial linker is not shown. B: Topology of HdaB‐dsA colored and labeled as in (A). C: Surface representation of HdaB‐dsA with self‐complementing donor strand from HdaA as sticks. Residues for interacting side‐chains in the HdaA strand are indicated.

**Figure 3:**
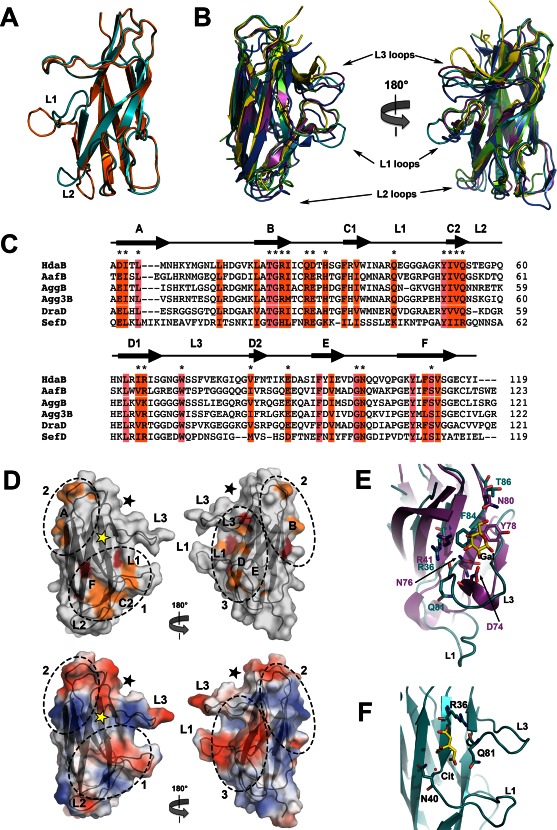
Putative functional binding regions of HdaB. A: Overlay of HdaB‐dsA from chains A and B. Regions that display significant structural variation are annotated. B: Cartoon representation of HdaB‐dsA (teal) superposed with DraD (pdb: 2axw in purple), AggB (pdb: 4phx in yellow), AafB (pdb: 2orl in green) and SefD (pdb: 3uiz in blue). Regions that display significant structural variation are annotated. C: Primary sequence alignment of HdaB (UniProtKB: B3V224), AafB (UniProtKB: D3H575), AggB (UniProtKB: P46006), Agg3B (UniProtKB: C9K5V1), DraD/AfaD (UniProtKB: Q47038) and SefD (UniProtKB: Q53997). Identical and similar amino acid residues are shaded in red and orange, respectively. Secondary structure of HdaB is shown above as lines (loops) and arrows (β‐strands), and * represents conserved residues that are exposed on the surface of HdaB. D: Upper panel: surface representation of monomeric HdaB‐dsA colored based on (C). Lower panel: electrostatic surface potential of HdaB‐dsA. Three regions with sequence conservation based on (C) are circled and labeled 1 to 3. Secondary structure within these regions are annotated as in Figure 2(B). The citrate 1/1′ binding site in HdaB_dsA is represented as a yellow star and the potential galactose binding site is shown as a black star. E: Potential binding site of galactose in HdaB. PsaA/galactose complex (pdb: 4f8p) is superimposed onto HdaB‐dsA and key residues are shown as sticks. F: Citrate 1 binding site on HdaB_dsA chain A with key residues are shown as sticks.

All HdaB‐dsA residues could be built into electron density maps except for the majority of the disordered N‐terminal vector encoded His_6_ tag and disordered Lys124 (chain A) from within the linker sequence. The final model also contains 318 water molecules, three citrate ions and a single iodide ion. As expected for a CU pilin domain, a single molecule of HdaB is formed from a classical Ig‐like fold, with the final G‐strand donated by the NTE of HdaA [Fig. 2(A,B)]. Within the HdaA NTE, residues Ala1, Ile3, Ala5, His7 and Val9 complement the P1‐P5 pockets of HdaB, respectively [Fig. 2(C)]. The overall structures of the two HdaB‐dsA chains are essentially identical, however, substantial deviations are observed within loops L1 and L2 with an RMSD of 1.1 Å over all Cα atoms [Fig. 3(A)].

### Putative functional regions of HdaB

As anticipated, tertiary structure comparisons using the Dali server^25^ identified the EAEC AAF/I and AAF/II tip subunits AggB and AafB as having significant structural homology (RMSD 1.2 Å and 1.4 Å, respectively)^8^ with HdaB‐dsA (Table 2). In addition, other minor pilin tip members of the Afa/Dr superfamily were also highlighted: the diffusely adherent *E. coli* (DEAE) AfaD/DraD protein (RMSD 1.8 Å)^28^, ^29^ and the *Salmonella enteritidis* fimbriae 14 (SEF14) SefD protein (RMSD 1.9 Å)^30^ (Table 2). The secondary structure elements of these proteins superpose with little deviation; however, variations are localized to regions within the L1, L2 and L3 loops of all four structures [Fig. 3(B)] and could be of significance because dynamic loop regions are often important for protein function^31^.

**Table 2:** Tertiary Structure Analysis of *HdaB‐dsA*

Protein name	PDB code	*Z* score	RMSD	Sequence ID (%)
DraD	2axw	17.5	1.8 Å over 108 equivalent Cα residues	46
AafB	4orl	15.4	1.4 Å over 118 equivalent Cα residues	47
AggB	4phx	15.1	1.2 Å over 114 equivalent Cα residues	57
SefD	3uiz	12.0	1.9 Å over 115 equivalent Cα residues	19
SafA	2co4	11.6	1.9 Å over 111 equivalent Cα residues	12
AfaE‐III	1ut2	10.2	2.4 Å over 113 equivalent Cα residues	15
PsaA	4f8p	9.0	2.2 Å over 116 equivalent Cα residues	12
CssA	4b9j	8.5	2.4 Å over 102 equivalent Cα residues	12
CssB	4b9g	8.3	2.8 Å over 104 equivalent Cα residues	14

*Z‐*score values taken from the DALI server^25^.

RMSD calculated using COOT^26^.

Sequence ID calculated using Clustal Omega^27^.

Whilst the function of the AAF/IV shaft forming subunit HdaA likely promotes host adhesion, as do the major components of AAF/I and AAF/II,^8^, ^12^ the role of the minor tip domain, HdaB, is not known. The function of the *E. coli* Afa/Dr fimbriae tip protein AfaD is an invasin, which can recognize host β1 integrin and lead to bacterial internalization.^20^, ^29^ The SEF14 tip protein SefD, and the AAF/I and AAF/II tip proteins AggB and AafB, respectively, have also been shown to promote host invasion and it is therefore likely that HdaB too carries out this role.^6^, ^32^ However, an AafB allele of 042 strain is non‐invasive^18^ and therefore the conditions under which, or the extent to which AAF invasins contribute to cellular uptake is unclear. Furthermore, AafB induces inflammation during EAEC infection^18^ and so these fimbriae tip proteins may also carry out other unique functions of which we are still not aware.

We next mapped the sequence conservation between HdaB, AafB, AggB, Agg3B, AfaD, and SefD onto the surface of the HdaB‐dsA structure [Fig. 3(C,D)]. With this approach we identified three clear regions with localized conservation and we speculate that these may encompass residues involved in promoting cellular invasion. The first region is localized to Qln43 within loop L1, the C2 β‐strand and Ser112 from the F β‐strand; the second is situated at the inter‐domain boundary within the A and B β‐strands and the intervening loop; and the third region is spread over loops L1 and L3, the D1 and D2 β‐strands, and the D2‐E and E‐F loops. Although region 3 is predominantly charged, region 2 and particularly region 1 contain significant hydrophobic surface, and this may indicate protein:protein interaction sites.

Other structures identified by the server DALI with Z‐scores above 8.0 were the FGL CU assembled *Salmonella* SAF major pilin domain SafA^33^ (RMSD 1.9 Å), *E. coli* AFA‐III major pilin domain AafE‐III^24^ (RMSD 2.4 Å), *Yersinia pestis* PSA pilin domain PsaA^34^ (RMSD 2.2 Å). and the *E. coli* CS6 pilus subunits CssA and CssB^35^ (RMSD 2.4 Å and 2.8 Å, respectively) (Table 2). Again, the Ig‐like fold of these structures overlay well with deviations generally observed in loop regions and additional secondary structure elements (not shown).

SafA and AafE‐III form the major component of the SAF pilus and AFA‐III pilus shafts, and whereas a ligand has not been identified for SafA, AafE‐III promotes adhesion to host cell surfaces through recognition of CEACAMs.^36^ However, comparison of interfacial AafE‐III residues from a nuclear magnetic resonance (NMR) spectroscopy derived model of an AafE‐III/CEACAM5^36^ complex with HdaB shows no similarity. The CS6 pilus is composed of alternating subunits of CssA and CssB, which each recognize their own host cell surface receptors,^35^ but no structural data is available for a ligand complex. PSA pili on the other hand are composed solely of repeating PsaA subunits, which bind β1‐linked galactosyl residues in glycosphingolipids and the phosphocholine group in phospholipids,^34^ and a PsaA/galactose complex has been obtained.^34^

Examination of this interface shows that several of the PsaA residues that coordinate galactose are either identical or similar to those in HdaB [Fig. 3(E)]. Superposition of PsaA with HdaB places galactose across the C1 and D2 β‐strands and L3 loop. PsaA residues Arg41, Asn76 and Asn80 create hydrogen bonds with galactose, and Arg36, Qln81, and Thr86 occupy these positions in HdaB. An additional PsaA interaction with galactose comes from Asp74 within the equivalent L3 region of HdaB, and due to the dynamic nature of this loop several HdaB residues with similar properties could take up this position. Finally in PsaA Tyr78 is packed against the galactose ring and in HdaB this is occupied by Phe84.

The structure of HdaB‐dsA reported here has three citrate ions bound from the crystallization solution [Fig. 1(B)]. Two of these, citrate 1 and 1′, bind to equivalent positions toward the dimer interface on the A and B chains, whilst the third is located at the C‐terminal pole of chain B. Citrate 1 and 1′ are bound by Arg36, and Qln81 in HdaB, which overlaps with the putative galactose binding site [Fig. 1(F)]. Although we have not been able to detect any significant interactions between HdaB‐dsA and galactose or citrate *in vitro* using NMR spectroscopy (data not shown), it could be that these are non‐native ligands with very weak affinities but are occupying real functional binding sites.^37^ Moreover, if this is a genuine ligand site, it is located in a region of HdaB that lacks sequence conservation with other members of the Afa/Dr superfamily and therefore may be unique to AAF/IV [Fig. 3(C,D)].

To test the validity of our observations, we are now screening carbohydrate arrays and carrying out mutational analysis of these sites in functional assays. Although further work is required to unravel the functional details of how HdaB promotes EAEC infection, our new structure of HdaB sheds some light here and may help in the development of new‐targeted strategies to combat future EAEC outbreaks.

## Materials and Methods

### Expression and purification

A donor strand complemented construct of HdaB was created by PCR containing residues 1–119 of *hdaB* at the N‐terminus, followed by a HMDNQEFIPLCQA linker and finally the HdaA residues 1‐10 at the C‐terminus. HdaB‐dsA was cloned into a pQE‐30 plasmid (Qiagen) containing a vector encoded N‐terminal His_6_ tag. This was transformed into *E. coli* BL21 (DE3) strain and grown at 37°C in LB. Expression was induced with 0.5 mM IPTG at OD600 nm of 0.6 and incubated for a following 4 hrs. Attempts to purify natively folded HdaB‐dsA were unsuccessful and therefore after harvesting the cells, they were lysed in the presence of 8 M urea and HdaB‐dsA was purified using Ni^2+^‐affinity chromatography under denaturing conditions^38^. After elution, HdaB‐dsA was dialyzed against 50 mM NaOAc pH 5.0, 200 mM NaCl, 1.0 M urea, 10 mM β‐mercaptoethanol followed by 50 mM NaOAc pH 5.0, 200 mM NaCl, then finally gel filtered with a Superdex‐75 column (GE healthcare) pre‐equilibrated in the same buffer.

### Crystallization, data collection, and structure determination

HdaB‐dsA (6 mg/ml) was crystallized using hanging‐drop vapor diffusion at 293K in 200 mM ammonium citrate pH 4.8, 20% (*w*/*v*) PEG 3350. Crystals were obtained after 2 weeks and then briefly soaked for 30 sec in this reservoir solution containing an additional 20% (*w*/*v*) PEG 3350, 0.5 M NaI and then flash frozen in liquid N_2_. Diffraction data were collected at 100 K on beamline I24 of the Diamond Light Source (DLS), UK. Data were processed using XDS^39^ and scaled with SCALA^40^ to 2.0 Å. Molecular replacement was performed with PHASER^41^ using the structure of AafB (pdb: 4OR1)^8^ as the search model. PARROT^42^ was used to remove model bias and automated model building was performed with BUCANEER.^43^ Refinement was carried out in REFMAC^44^ implementing TLS and NCS restraints, with 10% of the reflections omitted for cross‐validation. Manual model building was carried out in COOT.^26^ Processing and refinement statistics for the final model can be found in Table 1.

### Accession numbers

Coordinates and structure factors for HdaB‐dsA have been deposited in the Protein Data Bank (PDB code 5D55).
